# Endothelial Microparticle-Mediated Transfer of microRNA-19b Inhibits the Function and Distribution of Lymphatic Vessels in Atherosclerotic Mice

**DOI:** 10.3389/fphys.2022.850298

**Published:** 2022-05-09

**Authors:** Shi-Ran Yu, Yu-Xia Cui, Zi-Qi Song, Su-Fang Li, Chun-Ying Zhang, Jun-Xian Song, Hong Chen

**Affiliations:** Department of Cardiology, Center for Cardiovascular Translational Research, Beijing Key Laboratory of Early Prediction and Intervention of Acute Myocardial Infarction, Peking University People’s Hospital, Beijing, China

**Keywords:** atherosclerosis, lymphatic system, endothelial microparticles-mediated transfer of microRNA-19b (EMPmiR-19b), transforming growth factor beta receptor type II (TGF-βRII), lymphatic vessel

## Abstract

In recent years, the function of the lymphatic system in atherosclerosis has attracted attention due to its role in immune cell trafficking, cholesterol removal from the periphery, and regulation of the inflammatory response. However, knowledge of the mechanisms regulating lymphangiogenesis and lymphatic function in the pathogenesis of atherosclerosis is limited. Endothelial microparticles carrying circulating microRNA (miRNA)s are known to mediate cell–cell communication, and our previous research showed that miRNA-19b in EMPs (EMP^miR-19b^) was significantly increased in circulation and atherosclerotic vessels, and this increase in EMP^miR-19b^ promoted atherosclerosis. The present study investigated whether atherogenic EMP^miR-19b^ influences pathological changes of the lymphatic system in atherosclerosis. We first verified increased miR-19b levels and loss of lymphatic system function in atherosclerotic mice. Atherogenic western diet-fed ApoE^-/-^ mice were injected with phosphate-buffered saline, EMPs carrying control miRNA (EMP^control^), or EMP^miR-19b^ intravenously. The function and distribution of the lymphatic system was assessed via confocal microscopy, Evans blue staining, and pathological analysis. The results showed that lymphatic system dysfunction existed in the early stage of atherosclerosis, and the observed pathological changes persisted at the later stage, companied by an increased microRNA-19b level. In ApoE^-/-^ mice systemically treated with EMP^miR-19b^, the distribution, transport function, and permeability of the lymphatic system were significantly inhibited. *In vitro* experiments showed that miRNA-19b may damage the lymphatic system by inhibiting lymphatic endothelial cell migration and tube formation, and a possible mechanism is the inhibition of transforming growth factor beta receptor type II (TGF-βRII) expression in lymphatic endothelial cells by miRNA-19b. Together, our findings demonstrate that atherogenic EMP^miR-19b^ may destroy lymphatic system function in atherosclerotic mice by downregulating TGF-βRII expression.

## Introduction

Cardiovascular disease (CVD) remains the leading cause of death and disability, and the associated morbidity and mortality have been steadily rising around the globe (Roth et al., 2020). Atherosclerosis is known to be a major contributor to this growing burden of CVD. Although the specific pathophysiological mechanism of atherosclerosis remains controversial, it is now generally believed that atherosclerosis is a progressive, chronic, vascular inflammatory disease, that mainly affects large blood vessels (Libby, 2002; Libby et al., 2002). Data from numerous clinical and experimental studies support that both inflammation and abnormal lipid metabolism play critical roles in the formation of atherosclerotic lesions and the subsequent development of clinical complications (Libby et al., 2009; Ridker et al., 2017a; Ridker et al., 2017b). Thus, further exploration of the underlying mechanism of atherosclerosis is critical for identifying new therapeutic targets.

As an essential circulatory system, the lymphatic system runs in conjunction with the blood circulation and plays a vital role in maintaining body fluid homeostasis, regulating inflammation and immunity, and mediating lipid transport (Schwager and Detmar, 2019). Widespread lymphatic vessel networks and their draining lymph nodes as well as some lymph organs together make up the lymphatic system and accomplish their functions (Abouelkheir et al., 2017). Inflammation is greatly associated with lymphatic system. On one hand, lymphatic system reconstruction is seen in inflammatory diseases, with changes in both construction and function (Zheng et al., 2014). On the other hand, the lymphatic system functions in regulating the inflammatory response by draining large amounts of extravasated fluid to ensure tissue fluid homeostasis, while also transporting cells, macromolecules, and fluid to accomplish immune surveillance and so on (Skobe and Detmar, 2000; Vaahtomeri et al., 2017). Atherosclerosis as a vascular inflammatory disease, and the lymphatic system is likely to play a critical role in its pathophysiology (Kutkut et al., 2015). Recent data show the distribution of lymphatic vessels in both the intima and adventitia of arteries in dogs, humans and other animals. Additional studies revealed changes in the destination of lymphatic vessels associated with atherosclerotic vessels such as the human carotid arteries, and the changes of the number of lymphatic vessels is closely related to the severity of atherosclerosis. As for its function in atherosclerosis, the literature showing that lymphatic system is involved in many aspects of the course of atherosclerosis. With regard to dyslipidemia in atherosclerosis, research has indicated that lymphatic system insufficiency can alter lipoprotein levels and promote atherogenesis (Vuorio et al., 2014). Except taking part in the reverse cholesterol transport, some also found that lymphatic network could absorb and traffic cytokines and immune cells infiltrating in tissues to peripheral lymph node and then could mediate further inflammation response. Researchers found that improved the number of lymphatic vessels could relive tissues’ inflammatory mediators infiltration and alleviate high-fat diet mice vascular inflammation (Rademakers et al., 2017; Drosos et al., 2019; Milasan et al., 2019). All these findings reflect a strong connection between the lymphatic system to atherosclerosis. However, the mechanisms regulating dysfunction of the lymphatic system in atherosclerosis remain incompletely understood.

Extracellular vesicles (EVs) are important biological entities not only for cellular signaling in pathological progress but also as diagnostic and therapeutic tools. According to size, EVs are divided into three types: the largest is apoptotic bodies (ABs) with a size of 1–5 μm, the intermediate type is microparticles/microvesicles with a size of 100–1,000 nm, and the smallest is exosomes with diameters in the range of ∼40–100 nm (Loyer et al., 2014). Endothelial microparticles (EMPs) are released from vascular endothelial cells under basal or pathological conditions (Charla et al., 2020). Clinical data show that EMPs can serve as novel biomarkers, because most patients with atherosclerotic CVD have an elevated plasma EMP concentration (Chirinos et al., 2005). Further experimental data have revealed that increased EMP levels not only reflect the pathological processes of CVD, such as endothelial dysfunction and the development of atherosclerosis, but these particles also contain functional factors such as microRNAs, proteins and others and thereby mediate cell–cell crosstalk (Brodsky et al., 2004; Densmore et al., 2006). Our previous study results demonstrated that the level of microRNA-19b (miR-19b) encapsulated in EMPs may partially contribute to the upregulation of circulating miR-19b in patients with unstable angina (Li et al., 2014). Further animal experiments showed that systemic treatment with EMP^miR-19b^ significantly accelerated atherosclerosis via targeting of cytokine signaling 3 (SOCS3) expression. (Li et al., 2018). However, whether the EMP^miR-19b^ take part in regulating dysfunction of the lymphatic system in atherosclerosis remains unclear.

MiRNAs are known to be important mediators in the development and function of the lymphatic system. For example, miR-9/miR-1236 can stimulate lymphatic system activation in pathological conditions like inflammation. Targeting of miRNAs such as miR-126, miR-9, and miR-132 was also reported to be a potential therapeutic approach for correcting lymphatic system dysfunction. However, in chronic vascular diseases, including atherosclerosis, the potential of miRNAs as mediators for the distribution and function of lymphatic system remains unknown (Yee et al., 2017; Jung et al., 2019).

In present study we investigated whether miR-19b delivered by EMPs could affect the distribution and function of the lymphatic system during the atherosclerotic pathological process in an animal model. We then further explored the underlying mechanism for the observed effects of EMP^miR-19b^.

## Material and Methods

### Isolation and Characterization of EMPs From HUVECs

Human umbilical vein endothelial cells (HUVECs) were isolated from fresh human umbilical cord veins by collagenase I digestion according to the standard technique documented by Jaffe et al. (Takaoka et al., 2009). Isolated HUVECs were cultured with endothelial cell growth medium (ECM) with 5% fetal bovine serum (FBS), 1% penicillin and streptomycin, and 1% endothelial cell growth factor (EGF) under standard cell culture conditions (37°C, 5% CO_2_). HUVECs of passages 3–5 were used for experiments. Once HUVECs reached 70–80% confluence, they were transfected with miRNA control or miR-19b mimic (transfection concentration: 30 pmol/ml, GenePharma, China) using Lipofectamine 2000 (Invitrogen) for 24 h. Then the medium was changed to serum-free medium, and the cells were exposed to hypoxic conditions (37°C, 3% O_2_) for 12 h to generate modified EMPs. After cell collection, the production of EMP^control^ or EMP^miR-19b^ was performed as previously described (Jansen et al., 2013). Briefly, EMPs were isolated from HUVEC culture medium after centrifugation at 800 *g* for 15 min (to remove cells) and 12,500 *g* for 5 min (to remove debris). The supernatant was further centrifuged at 4°C at 20,500 *g* for 2.5 h. EMPs were collected from the sediment, and standard assessment of isolated EMPs was performed as previously described (Li et al., 2018).

### Animals and Procedures

All animal experiments were approved by the Medical Ethics Committee of Peking University People’s Hospital and carried out according to the National Institutes of Health guide for the care and use of laboratory animals (NIH Publications No. 8023, revised 1978). Six-to eight-week-old male ApoE^-/-^ mice were provided by Peking University Experimental Animal Center and were housed in pathogen-free barrier facilities under appropriate conditions with a temperature of 22 ± 2°C, humidity of 55 ± 5%, and 12-h light/12-h dark time cycle. After a 1-week acclimatization period with normal chow, mice were divided into two groups, one fed normal chow and the other fed a high-cholesterol atherogenic western diet (21% fat and 0.15% cholesterol, HFK Bioscience, China). The mice fed the high-cholesterol atherogenic diet were further subdivided into three groups that received injection of phosphate-buffered saline (PBS) as a control or different types of EMPs (1 × 10^7^ EMPs) through the tail vein three times per week throughout the experiment according to previous protocol (Li et al., 2018). In our study, we also monitored the miR-19b level in blood to guarantee the levels in EMP^miR-19b^ mice were higher than those in the control groups. These three groups included the PBS group (injected with PBS, as a blank control), the EMP^control^ group (injected with EMP^control^, as a negative control), and the EMP^miR-19b^ group (injected with EMP^miR-19b^). EMPs were injected over an 8-week study period, and blood, lymph and aortic arch tissues were collected at 4, 6, and 8 weeks.

### Carotid Ultrasonography

For imaging of carotid arteries, the mice were anesthetized, and the hair was shaven from the anterior cervical skin. Ultrasound imaging was performed with mice in the supine position using a high-frequency ultrasound system (Vevo2020, VisualSonics). Carotid aorta pulse wave velocity (PWV) was determined with a 24-MHz transducer following the instructions of the device manufacturer. Briefly, pulse wave Doppler tracing was used to measure aortic flow velocity (V). Immediately thereafter, the aortic diameter (D) in the systolic and diastolic stages was measured on 700 frames-per second B-mode images of the carotid aorta in the EKV imaging mode. The carotid resistive index (RI) was calculated according to the peak systolic velocity (PSV) and end-diastolic velocity (EDV), using the formula: RI = [(PSV-EDV)/PSV].

### Histological Evaluation of Atherosclerotic Lesions

The aortic arch tissues were fixed in 4% paraformaldehyde for more than 24 h, embedded in paraffin, and cut into 4.5-μm-thick sections. The sections were stained with hematoxylin and eosin (H&E) and Masson’s trichrome staining.

### Immunofluorescence Staining

The 4.5-μm-thick paraffin-embedded aortic arch tissue sections were deparaffinized, and nonspecific reactions were blocked with goat serum for 15 min at 37°C. Then, the sections were incubated with primary polyclonal antibodies overnight at 4°C. The primary antibodies used included lymphatic vessel endothelial hyaluronan receptor-1 (LYVE1) mouse monoclonal antibody (Proteintech, United States) and CD68 rabbit polyclonal antibody (Proteintech). The following secondary antibodies were used for detection: goat anti-mouse immunoglobulin G (IgG) H&L (Alexa Fluor^®^ 488) and donkey anti-rabbit IgG H&L (Alexa Fluor^®^ 647) (both from Abcam, USA). Sections were counterstained with 4,6-diamidino-2-phenylindole (DAPI) for visualization of cell nuclei and mounted for analysis.

### RNA Extraction and Quantitative Real-Time PCR

Total RNA was isolated from the aortic tissues, lymph, and lymphatic endothelial cells (LECs) using TRIzol Reagent (Thermo Fisher Scientific, United States). Briefly, add 750 µl TRIzol Reagent in each sample, all of them are equal in volume or weight, and exogenous cel-miRNA 39 was added in lymph samples as an internal control. Completely dissociated tissues and cells were incubated with trichloromethane with one-fifth of the volume of TRIzol after 5 min. The samples were mixed thoroughly and then centrifuged at 12000 *g* for 15 min at 4°C. An equal volume of isopropanol was added to the supernatant, and after centrifugation, the RNA was purified with anhydrous ethanol. Finally, the extracted RNA was diluted in diethylpyrocarbonate (DEPC)-treated water, and the concentration was determined. Reverse transcription quantitative real-time PCR (RT-PCR) for determination of mRNA and miRNA expression was performed on a LightCycler Run 5.32 Real-Time PCR System (Roche, Swiss) using SYBR Green detection chemistry and Taqman Universal Mix II. All samples were quantitated by the comparative CT method for relative quantitation of gene expressions. The expression of *gapdh* was used as the mRNA internal control, whereas U6 and miNA-39 were used as the miRNA internal controls for tissues and lymph, respectively. The following primer sequences were used: homo-*tgfβRII*: forward 5′-GCT TTG CTG AGG TCT ATA AGG C-3′, reverse 5′-GGT ACT CCT GTA GGT TGC CCT-3'; homo-*gapdh*: forward 5′-GAG TCA ACG GAT TTG GTC GT-3′, reverse 5′-GAC AAG CTT CCC GTT CTC AG -3'. The samples were analyzed in triplicate.

### Collection of Lymphatic Fluid From the Cisterna Chyli

For lymphatic fluid collection, the mice were fasted for 12–14 h. At 1 h before collection, 200 μl soybean oil was administered by gavage to mice to make the cisterna chyli more visually obvious. The mice were anesthetized, and the abdomen was opened horizontally. Lymphatic flow was confirmed by the white lucent color in the intestinal trunk and the cisterna chyli under stereomicroscopy. The intestinal trunk is formed by the confluence of the efferent vessels of the cranial mesenteric and celiac nodes, and the single trunk enters into the cisterna chyli located along the abdominal vena cava and aorta on the cranial side of the renal veins. The cisterna chyli was cut with the bevel of a fine needle, and the lymphatic fluid was collected with a 24G catheter needle connected to a glass tube moistened with heparin. The lymphatic fluid was finally collected in an Eppendorf tube moistened with heparin for anticoagulation. The collected lymph was centrifuged at 1000 rpm for 5 min to generate a pellet of the cells present in the lymph. Then the supernatant was removed and saved for further analysis.

### Lymphatic Vessel Permeability

Evans blue dye was used to assess lymphatic vessel permeability through tracing the path of lymph through popliteal lymphatic vessels. Mice were anesthetized by isoflurane inhalation anesthesia, and skin was carefully removed from the legs. Following Evans blue intradermal injection in the footpad, popliteal collecting lymphatic vessels were visualized under a Leica M320 operating microscope (Leica). The effusion of Evans blue around the vessels marked the area of the leakage and was analyzed using ImageJ software (National Institutes of Health, United States).

### Molecular Transport Within Lymphatic Vessels

Lymphatic vessel transport of molecular substances was assessed following injection of fluorescein isothiocyanate (FITC)-labeled dextran in the dermis of the footpad of the mouse (Fukasawa et al., 2021). After injection, we continuously detected the fluorescence intensity change in the popliteal lymph node by confocal microscopy (Leica SP5). Briefly, the mouse was placed in prone position on a heating pad at 40°C, and lower limb skin was removed, ensuring no blood vessels were severed as the skin was pulled away. Warm saline was administered to keep the limb tissue hydrated at all times. We first identified the location of lymph nodes under brightfield microscopy, and then 50 μl FITC-dextran (5 mg/ml) was injected in the footpad (Fukasawa et al., 2021). Once perfusion of the tracer appeared, the lymph node was recorded for 45 min to continuously observe the fluorescence intensity change in the popliteal lymph node from the first appearance of fluorescence until the fluorescence intensity declined. To quantify the fluorescence intensity, we traced a line of equivalent length at three different regions of interest (ROIs) along the lymph node, and the average value was used for analysis.

### Cell Culture and Transfection

Primary human dermal lymphatic endothelial cells (HDLECs, Promocell) were cultured according to the manufacturer’s protocol (Lonza) in EBM-2 medium containing EGM-2 MV SingleQuots. Cells of passage 5–6 were seeded in culture dishes at 80% confluence and transfected with miRNA control, miR-19b mimic, miRNA inhibitor control, or miR-19b inhibitor (GenePharma, China) using Lipofectamine 2000 (Invitrogen) for 24 h.

### Migration Assay

The cell migration assay was performed using 24-well Transwell chambers. HDLEC suspension (100 μl ECMV2 without FBS) from each group was added to the upper chamber (1 × 10^5^ cells/well), whiles 600 μl ECMV2 with 5% FBS was added to the bottom chamber. After 12 h of incubation at 37°C, cells on the upper membrane surfaces were removed, and migrated cells were washed three times with PBS at 37°C. The migrated cells were fixed with 4% paraformaldehyde for 15 min and stained with crystal violet for 20 min. The number of migrated cells was determined by counting in five fields with a uniform cell distribution under an inverted microscope. The assay was repeated three times, and the average migration rate was calculated.

### Tube Formation Assay

For use in the tube formation assay, Matrigel was thawed at 4°C overnight. Then each well of a prechilled 48-well plate was coated with 150 μl Matrigel and incubated for 1 h at 37°C in a 5% CO_2_ incubator. Then, 200 μl cell suspension was added to each well (2 × 10^5^ cells/well). After incubation of the different groups of cells under specified culture conditions for 4–6 h, tube-like structures and their arrangement, amount, and degree were evaluated in photographs taken of five randomly selected five fields under a microscope. The tube number was quantified using ImageJ software.

### Statistical Analysis

Data are expressed as the mean ± standard error of the mean (SEM). Statistical significance was evaluated by unpaired *t*-test or, for multiple comparisons, one-way analysis of variance (ANOVA) using appropriate corrections when data were not normally distributed or for unequal variances. All calculations were done with GraphPad Prism software (GraphPad Software, La Jolla, CA, United States), and *p* values < 0.05 were considered statistically significant.

## Results

### Lymphatic Vessel Dysfunction Occurs in the Early Stage of Atherosclerosis

**FIGURE 1:**
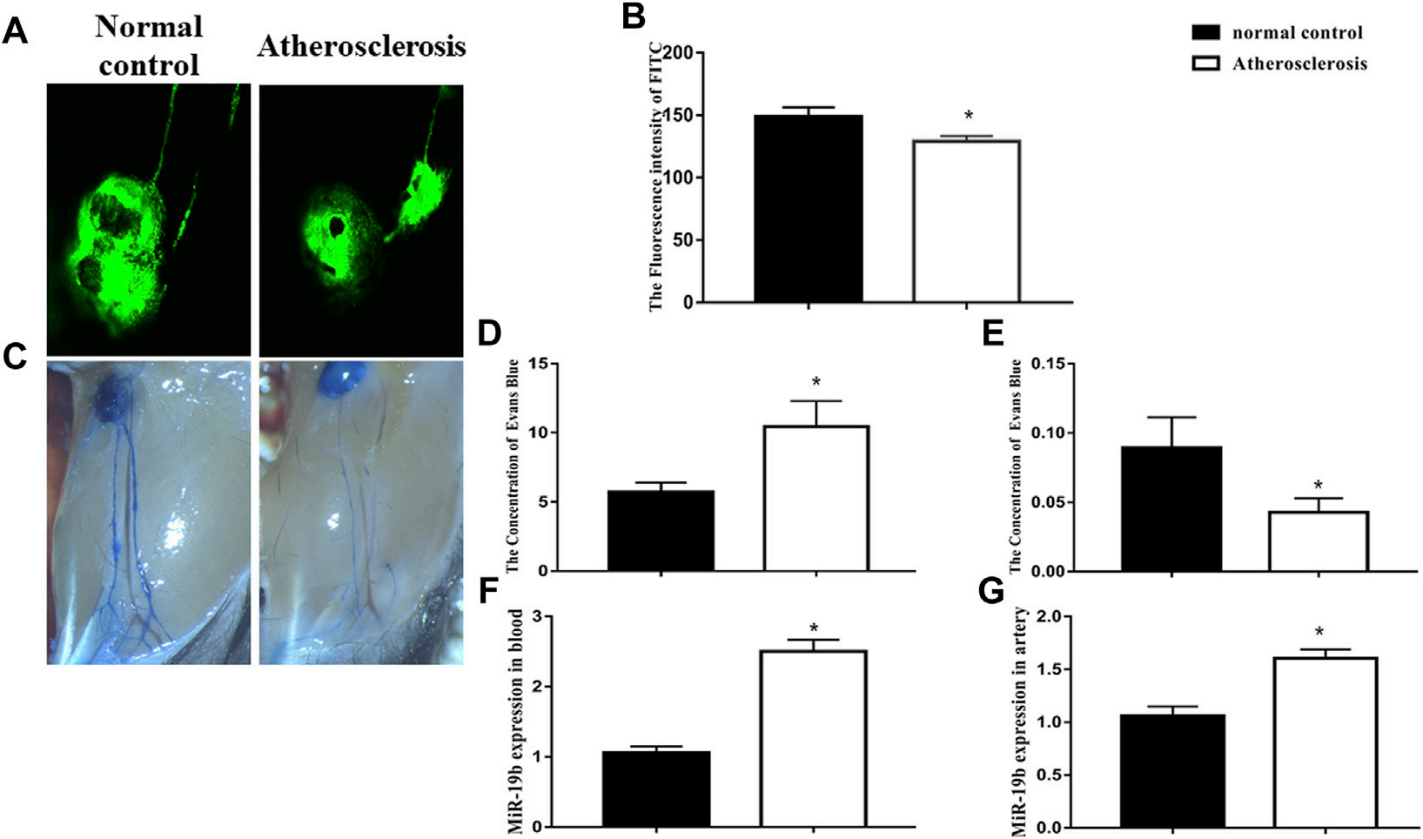
Lymphatic vessel dysfunction occurs in the early stage of atherosclerosis. **(A)** Representative confocal image of FITC fluorescence intensity in popliteal lymph nodes from normal diet-fed and high-fat diet-fed ApoE^-/-^ mice. **(B)** Quantitative analysis of the results shown in **(A)** for the normal control and atherosclerosis groups. *p < 0.05 compared with normal control group, *n* = 7 per group. **(C)** Representative Evans blue staining images of lymphatic vessels in legs of normal diet-fed and high-fat diet-fed ApoE^-/-^ mice after 4 weeks of feeding. **(D)** Amount of Evans blue dye retained in the injected leg lymph node at 10 min after injection. **(E)** Amount of Evans blue dye retained in opposite leg lymph node at 10 min after injection. *p < 0.05 compared with normal control group, *n* = 7 per group. **(F–G)** RT-PCR results for the expression of miRNA-19b in blood and atherosclerotic artery tissue.

We first observe pathological changes in the lymphatic system of atherosclerotic mice after 4 weeks of high-fat diet feeding. The transport function of lymphatic vessels was measured via confocal microscopy, and the permeability of lymphatic vessels was assessed by Evans blue staining in each group. Fluorescence images and videos revealed that, upon injection, FITC-labeled dextran was taken up by peripheral lymphatic vessels of the legs and then drained into the popliteal fossa lymph nodes. Comparison of the different groups showed that the time to reach maximum intensity in high-fat diet-fed mice was significantly extended and the maximum intensity significantly reduced compared with those in mice fed the control diet (*p* < 0.05, Figure 1A). The results of Evans blue staining revealed that the lymphatic vessels in the atherosclerotic mice were thinner, and exudation of blue dye from these vessels was increased compared with the control (Figure 1C). Next, the residual amount of Evans blue dye and the amount of Evans blue dye in the opposite leg lymph node were measured at 10 min after injection. The results showed that the amount of residual dye in atherosclerotic mice was greater than that in mice fed a normal diet (Figure 1D), whereas the amount of Evans blue dye in the opposite leg lymph node was less than that in the normal diet-fed mice (Figure 1E). Both confocal microscopy and Evans blue staining indicated that the transport function and permeability of the lymphatic system were disrupted in atherosclerotic mice after 4 weeks of high-fat diet feeding.

We also detected the level of miRNA-19b in aortic tissue and blood. Quantitative PCR analysis revealed that the level of miRNA-19b was increased in the aortic tissue and blood of high-fat diet-fed mice compared with normal diet-fed mice (Figures 1F,G).

### EMP^miR-19b^ Promotes the Development of Atherosclerosis in ApoE^-/-^ Mice

**FIGURE 2:**
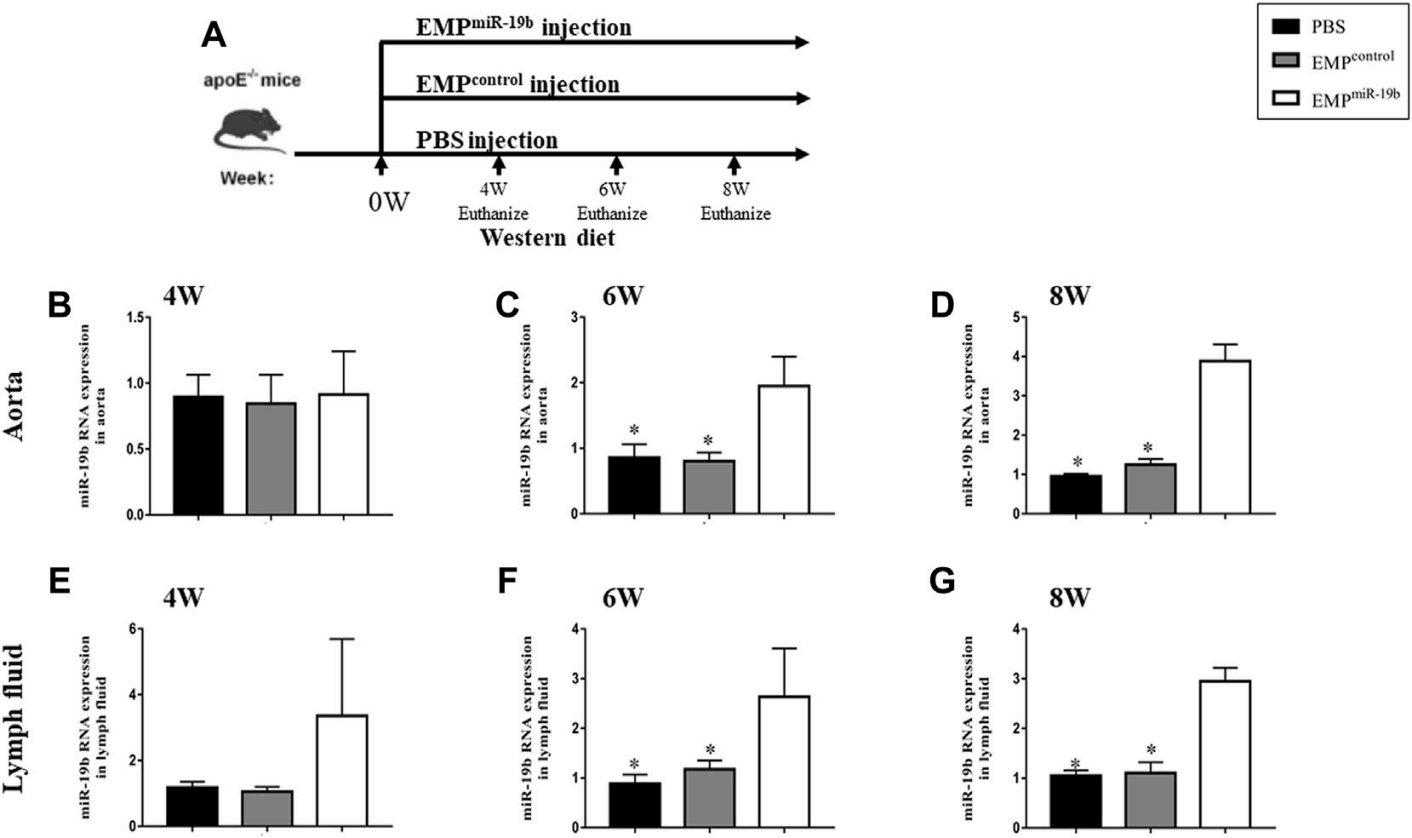
miR-19b levels in different tissues from mice in the three treatment groups after 4, 6, and 8 weeks of treatment. **(A)** Schematic of the experimental procedure for EMP or contrl treatment and different observation points. **(B)** After 4 and 6 weeks **(C)** and 8 weeks **(D)** of treatment, miR-19b levels in aortic tissue of mice injected with the indicated EMPs. *p < 0.05 compared with EMP^miR-19b^ group, *n* = 7–8 per group. **(E)** After 4 and 6 weeks **(F)** and 8 weeks **(G)** of treatment, miR-19b levels in lymph fluid of mice injected with the indicated EMPs. *p < 0.05 compared with EMP^miR-19b^ group, *n* = 7–8 per group.

To investigate the role of miR-19b-containing EMPs in the development of atherosclerosis, 6-week-old ApoE^-/-^ mice fed a high-fat diet were treated with PBS, EMP^control^, or EMP^miR-19b^ three times per week by tail vein injection (Figure 2A). After the 4, 6, or 8 weeks of treatment, miR-19b levels in lymph and aortic tissue samples were measured. The RT-PCR results show that in each sampling point, the miR-19b levels in both aortic tissue samples (Figures 2B–D) and lymph fluid (Figures 2E–G) were elevated in EMP^miR-19b^-treated mice compared with PBS- and EMP^control^-treated controls, indicating that systemic EMP^miR-19b^ treatment could increase miR-19b expression in lymphatic circulation and arterial tissue at the same time.

**FIGURE 3:**
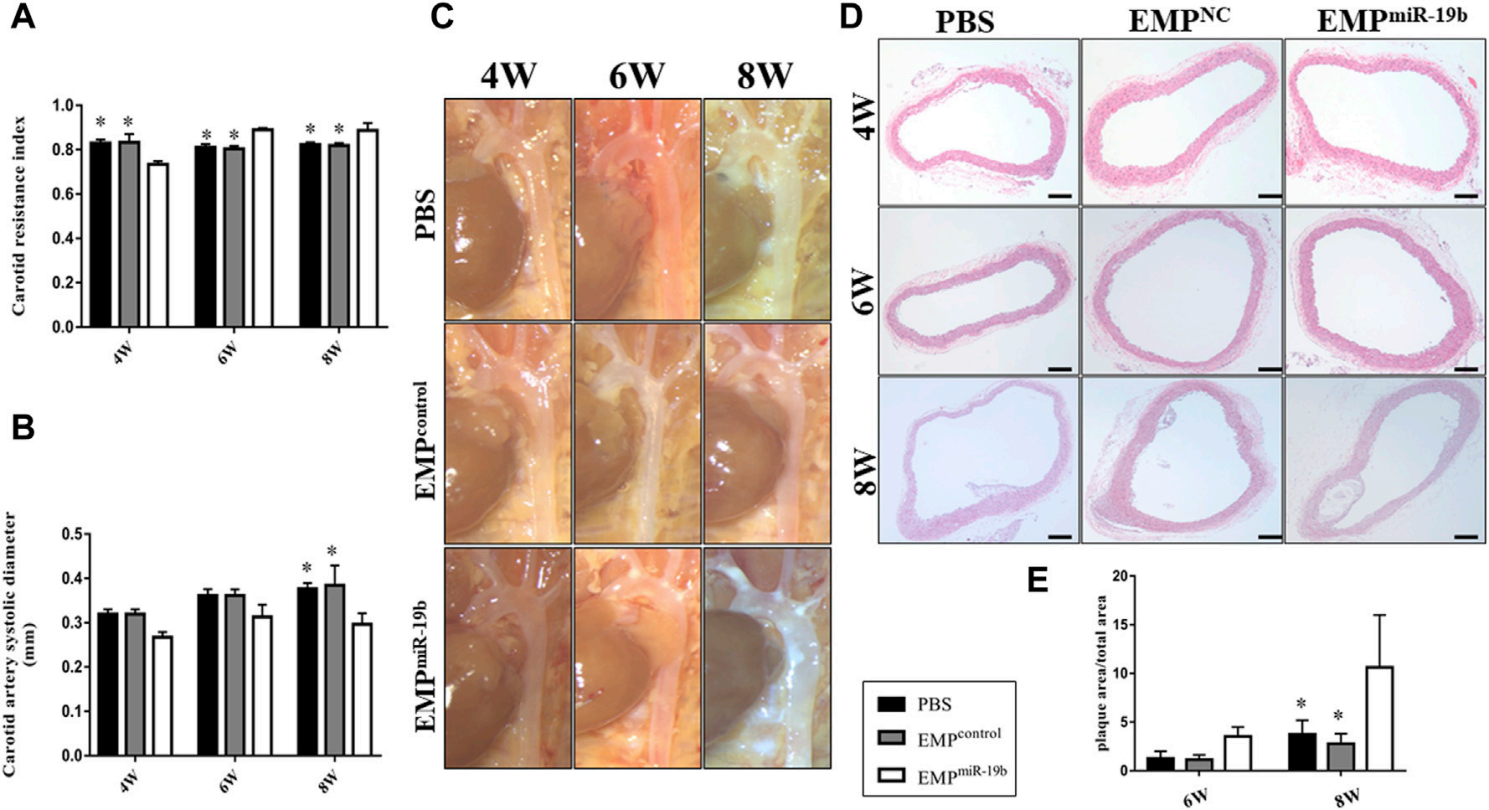
EMP^miR-19b^ promotes atherosclerotic plaque formation in ApoE^−/−^ mice. **(A)** Carotid ultrasound was used to assess arterial flow velocity (V) of carotid artery. The peak systolic velocity (PSV) and end-diastolic velocity (EDV) were measured and used to calculated the resistive index (RI) = (PSV–EDV)/PSV, as a flow parameter reflecting vascular resistance and vascular compliance. **(B)** Aortic diameter in systolic stage as a measure of artery size. **(C)** Gross pathological specimen from high-fat diet-fed ApoE^-/-^ mice intravenously injected with PBS, EMP^control^ or EMP^miR-19b^ for 4, 6, or 8 weeks. Representative images are presented. **(D)** Representative images of H&E staining of aortic sections from high-fat diet-fed ApoE^−/−^ mice intravenously injected with PBS, EMP^control^, or EMP^miR-19b^ for 4, 6, or 8 weeks. **(E)** Quantitative morphometric analysis of lesion area in **(D)**. *p < 0.05 compared with EMP^miR-19b^ group, *n* = 7–8 per group.

We then further assessed the effect of increased miR-19b expression on the pathological process of atherosclerosis. Firstly, carotid ultrasound showed that after 4, 6, and 8 weeks of treatment, the RI of the carotid artery in the EMP^miR-19b^ group mice was significantly elevated compared with that in the other control groups (Figure 3A). Meanwhile, the diameter of the carotid artery in the systolic stage showed a decreasing trend in the EMP^miR-19b^ group after 4 and 6 weeks of treatment. Then after 8 weeks of treatment, compared with the PBS- and EMP^control^-treated groups, the EMP^miR-19b^ group had a significantly decreased carotid artery diameter in the systolic stage (Figure 3B). All of the ultrasonography results strongly suggested that EMP^miR-19b^ accelerated the development of atherosclerosis.

The results of histopathological analyses supported the findings from imaging examinations. After 6 weeks of high-fat diet feeding, atherosclerotic plaque began to form in the aorta, especially in the aortic arch. After 6 weeks of PBS or EMP treatment, atherosclerotic plaque had formed in the aortic arch in all of three treatment groups; however, in the EMP^miR-19b^ group, the size of the plaque was larger and the distribution of plaque was much more extensive compared with results for the other groups, and these differences were even clearer after 8 weeks of treatment (Figure 3C). Cross-sectional analysis revealed that compared with PBS and EMP^control^ treatment, EMP^miR-19b^ treatment significantly increased the size of H&E-stained atherosclerotic plaque and the composition of plaque was more complex with a thinner fibrous cap and profoundly increased lipid accumulation (Figures 3D,E, *p* < 0.05). Together, the results of histopathological and ultrasound examinations suggested that treatment with EMP^miR-19b^ promoted the development of aortic atherosclerosis.

### EMP^miR-19b^ Damage Lymphatic Vessel Function in Atherosclerotic Mice

**FIGURE 4:**
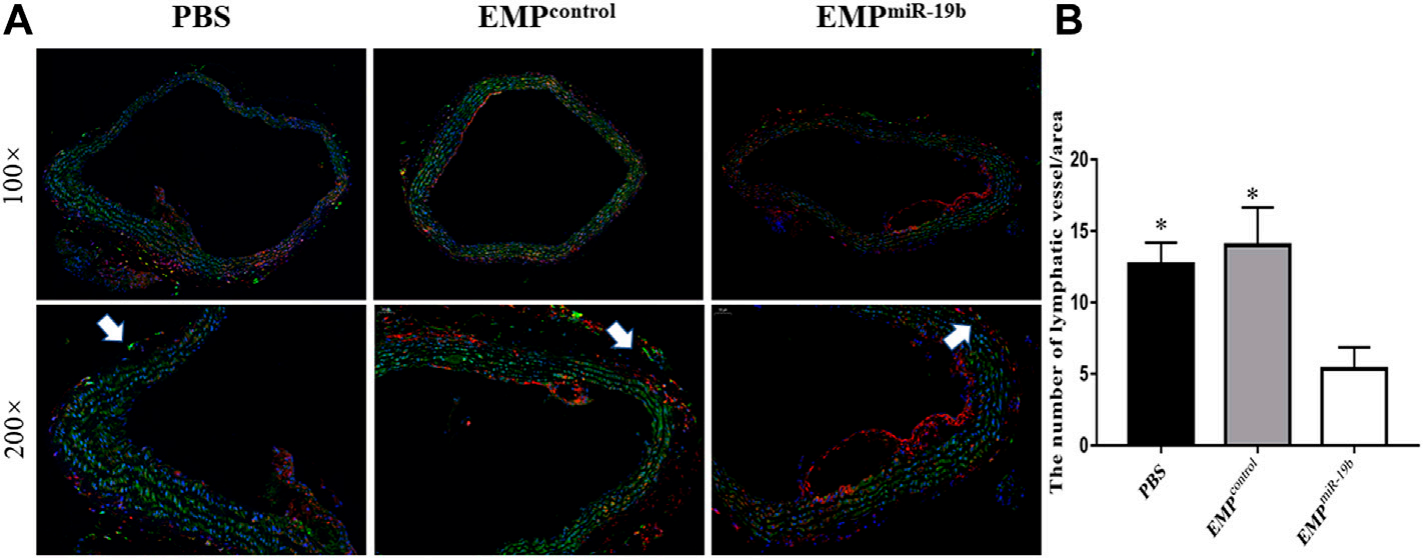
EMP^miR-19b^ limits lymphatic network distribution along atherosclerotic arteries. **(A)** Representative immunofluorescence staining images of atherosclerotic arteries from high-fat diet-fed ApoE^-/-^ mice intravenously injected with PBS, EMP^control^, or EMP^miR-19b^ for 8 weeks. In the images, green fluorescence represents LYVE1, a relatively specific marker of lymphatic vessels, while red fluorescence represents CD68. LYVE1^+^/CD68^-^ tubular structures are identified as lymphatic vessels. **(B)** Quantitative analysis of the results shown in **(A)**. *p < 0.05 compared with EMP^miR-19b^ group, *n* = 7–8 per group.

To understand whether EMP^miR-19b^ treatment could impact the function of lymphatic system, we further evaluated the transport function and permeability of lymphatic vessels. We first tested whether the lymphatic trafficking capacity was damaged by EMP^miR-19b^ treatment. Based on the current literature, we decided to inject FITC-labeled dextran into the foot pad of the mice and continually monitor the dynamic change in fluorescence intensity in popliteal fossa lymph nodes by confocal microscopy (Figure 4A). Fluorescence imaging revealed that, upon injection, FITC-labeled dextran was taken up by peripheral lymphatic vessels of the legs and then drained into the popliteal fossa lymph nodes. The results showed that the fluorescence intensity increased over time in both lymph nodes; however, at each observation time point, the time to reach maximum intensity in the EMP^miR-19b^ treatment group was progressively extended and the maximum intensity significantly reduced, with significant differences compared with the control groups (*p* < 0.05). The variation in the differences observed between the EMP^miR-19b^ group and the two control groups at the 4- and 6-week points indicate that lymphatic system dysfunction occurs in the early stages of disease.

To assess lymphatic vessel permeability, Evans blue dye was also injected in the mouse footpad, and then dye leakage and the size of the lymphatic vessels were quantified. Our results revealed that EMP^miR-19b^ treatment had no significant effect on dye leakage, but lymphatic vessel thickness was decreased after EMP^miR-19b^ treatment, compared with the thicknesses in the control groups (Figure 4E).

Both confocal microscopy and Evans blue staining results indicated that EMP^miR-19b^ treatment damaged the function of the lymphatic system in ApoE^−/−^ mice.

### EMP^miR-19b^ Inhibits Distribution of the Lymphatic Network With Atherosclerotic Arteries

**FIGURE 5:**
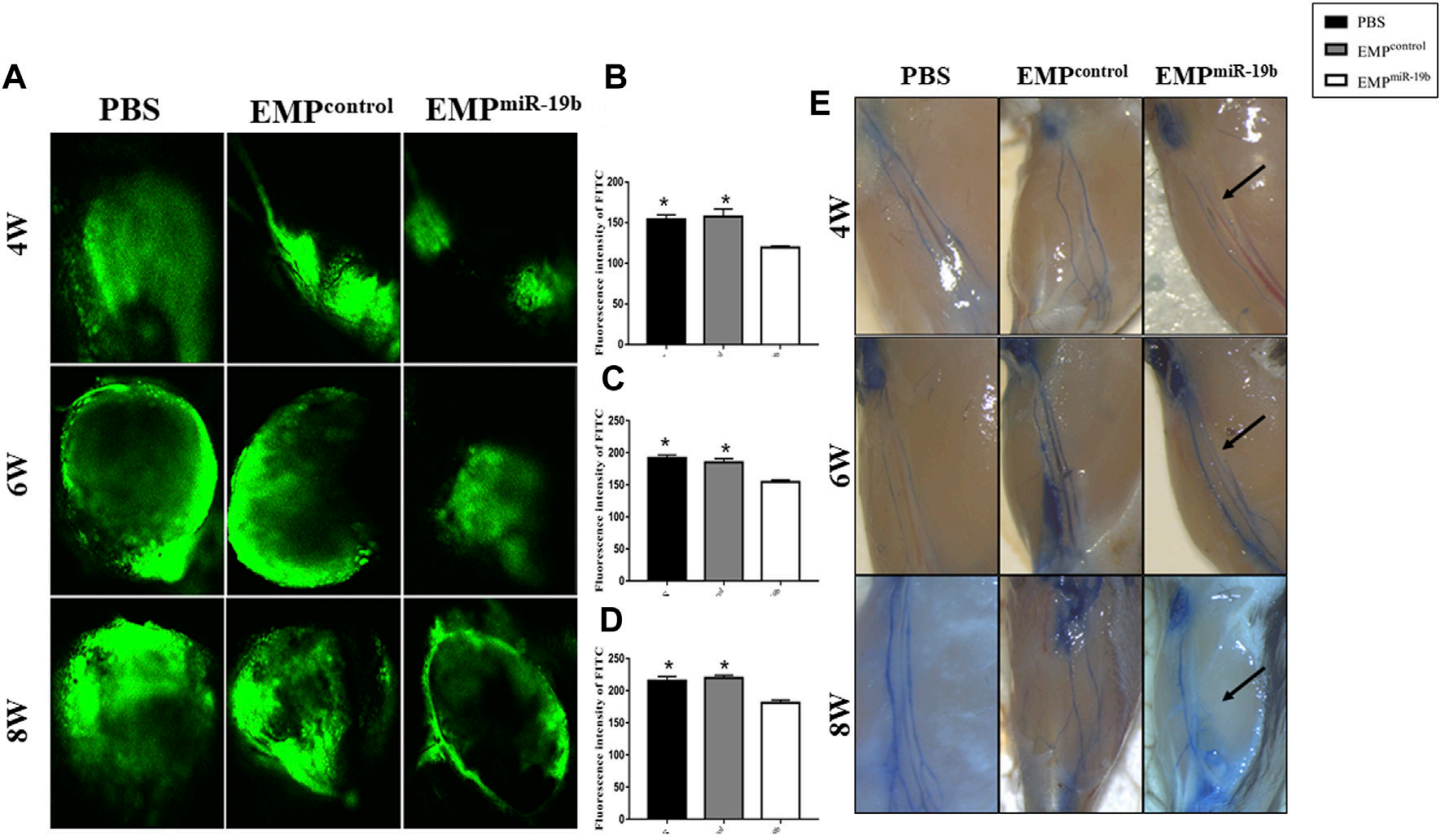
EMP^miR-19b^ induce lymphatic system dysfunction in ApoE^-/-^ mice. **(A)** Representative confocal images of FITC fluorescence intensity in popliteal lymph nodes of high-fat diet-fed ApoE^-/-^ mice intravenously injected with PBS, EMP^control^, or EMP^miR-19b^ for 4, 6, or 8 weeks. **(B–D)** Quantitative analysis of the results shown in **(A)**. *p < 0.05 compared with EMP^miR-19b^ group, *n* = 5–6 per group. **(E)** Representative images of Evans blue staining of lymphatic vessels in legs from high-fat diet-fed ApoE^-/-^ mice intravenously injected with PBS, EMP^control^, or EMP^miR-19b^ for 4, 6, or 8 weeks.

To understand whether EMP^miR-19b^ treatment could impact the distribution of lymphatic vessels along atherosclerotic arteries, we applied immunofluorescent staining for LYVE1, a relatively specific marker for lymphatic vessels, to observe lymphatic vessels with arteries in the three groups after 8 weeks of treatment (Figure 5). CD68 staining also was performed, because some CD68^+^ macrophages can also express LYVE1 to interfere with the results. Thus, in the double immunofluorescence staining images, the LYVE1^+^/CD68^-^ tubular structures are identified as lymphatic vessels (Schlereth et al., 2014). Fluorescence images revealed fewer lymphatic vessels with atherosclerotic arteries in the EMP^miR-19b^ group than in the two control groups (*p* < 0.05). This finding suggests that EMP^miR-19b^ treatment may inhibit the distribution of lymphatic vessels in the pathogenesis of atherosclerosis.

### miRNA-19b Inhibits LEC Migration and Lymphangiogenesis

**FIGURE 6:**
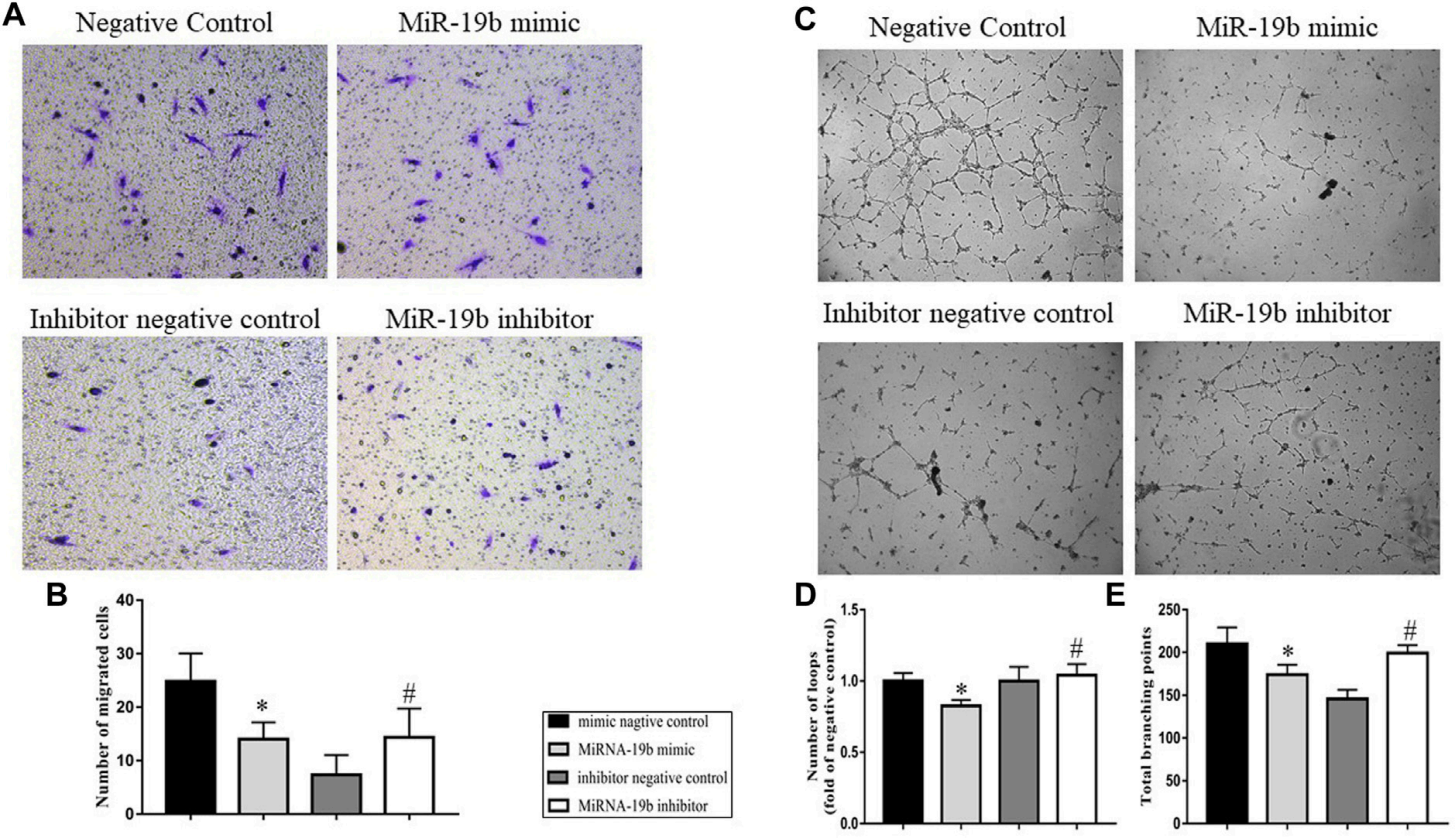
Effects of miR-19b on LEC migration and lymphangiogenesis. LECs were respectively transfected with miR-19b mimic (miR-19b mimic group) or a negative control fragment (negative control group), as well as miR-19b inhibitor (miR-19b inhibitor group) or a corresponding negative control (inhibitor negative control group). **(A)** Cell migration was assessed by Transwell migration assay and **(B)** quantified by measuring the number of migrated LECs; *n* = 3. **(C)** *In vitro* lymphangiogenesis was analyzed by tube formation assay and quantified by measuring the total number of **(D)** branching points and **(E)** loops; *n* = 3. Magnification, ×100. *p < 0.05 compared with negative control group, ^#^ *p* < 0.05 compared with the negative control group.

To further understand the effect of miRNA-19b on the lymphatic system, *in vitro* cell experiments were carried out. Transwell migration and tube formation assays were performed to examine the effects of miR-19b on LEC migration and lymphangiogenesis, respectively. In the Transwell migration assay, comparison of the numbers of migrated cells in each group showed that miR-19b inhibited LEC migration from the upper chamber to the reverse side. However, miR-19b inhibitor promoted LEC migration compared with the negative control (Figures 6A,B, *p* < 0.05). The results of the tube formation assay showed that the number of total branching points in the miR-19b mimic group was significantly less than that in the negative control group, and the number of loops formed was reduced by 0.8-fold compared to the control group. In contrast, the number of total branching points in the miR-19b inhibitor group was significantly greater than that in the corresponding negative control group, and the number of loops formed was increased by 1.2-fold as compared to the control (Figures 6C–E, *p* < 0.05).

### miRNA-19b Inhibits TGFβRII Expression in LECs

**FIGURE 7:**
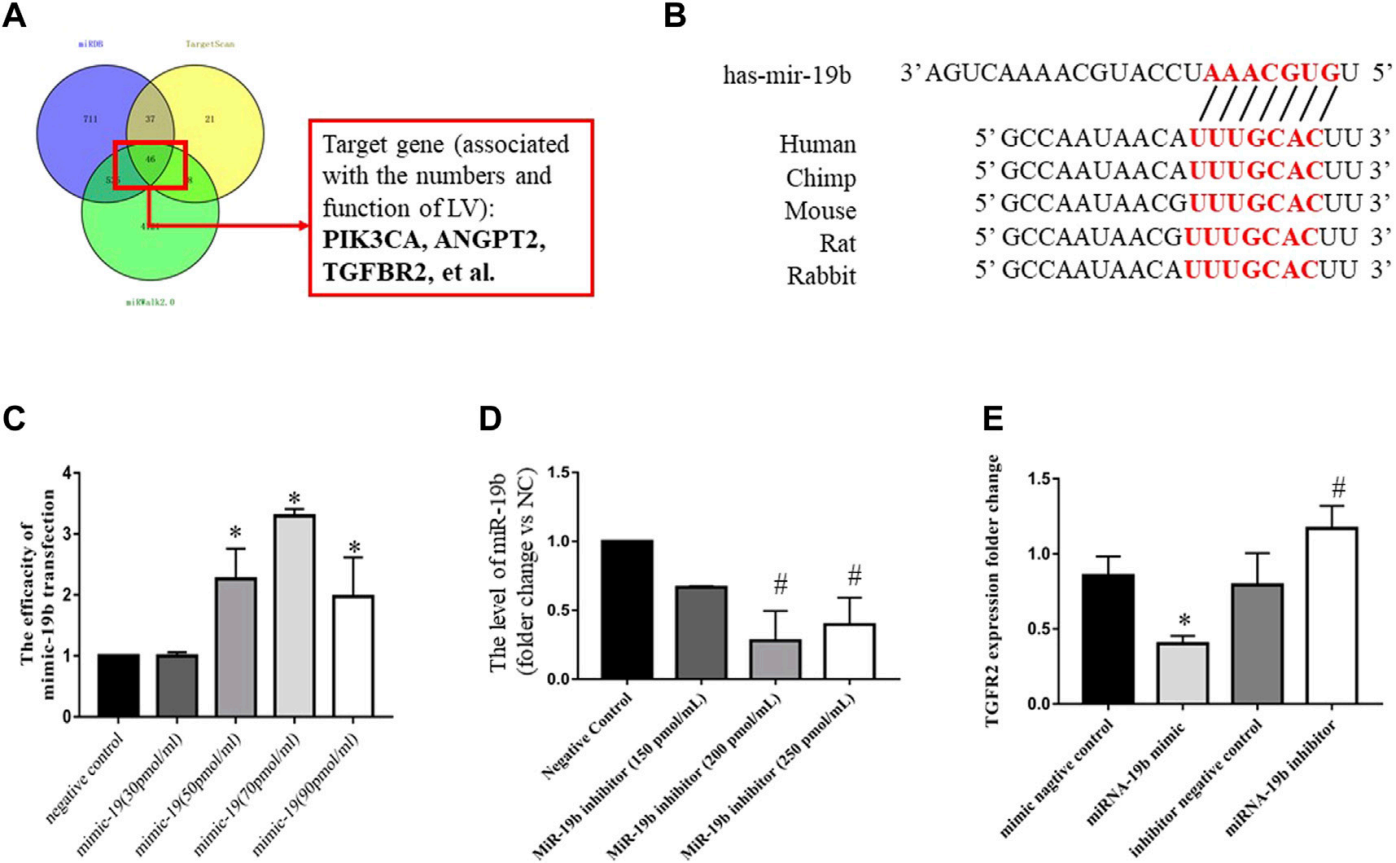
Regulatory effects of miR-19b on TGFβRII expression in LECs. **(A)** Schematic of miRNA-19b target prediction. **(B)** Schematic representation of the 3′ untranslated region of TGFβRII mRNA with the predicted target sites for miR-19b as well as miR-19b inhibitor (miR-19b inhibitor group) and a corresponding negative control (inhibitor negative control group). **(C)** LECs were transfected with miR-19b mimic at different concentrations or a negative control fragment, and miR-19b expression was measured by RT-PCR and normalized to RNU6B expression. *p < 0.05 compared with the respective control group. **(D)** LECs were transfected with miR-19b inhibitor at different concentrations and a corresponding negative control fragment, and miR-19b expression was measured by RT-PCR and normalized to RNU6B expression. ^#^ *p* < 0.05 compared with negative control. **(E)** LECs were transfected with miR-19b mimic (miR-19b mimic group) or a negative control fragment (negative control group) as well as miR-19b inhibitor (miR-19b inhibitor group) or a corresponding negative control (inhibitor negative control group), and TGFβRII mRNA expression was measured in the different groups. *p < 0.05, ^#^ *p* < 0.05 compared with the respective control group.

To identify a potential target gene of miR-19b associated with the distribution and function of lymphatic vessels, we performed an in silico analysis using three different microRNA target prediction algorithms, TargetScan (http://www.targetscan.org/), miRDB (http://mirdb.org/), and miRWalk2.0 (http://zmf.umm.uni-heidelberg.de/apps/zmf/mirwalk2/index.html). The results identified TGFβRII as a potential target commonly predicted by the three algorithms (Figures 7A,B).

To determine whether TGFβRII is a novel target of miR-19b, gain- and loss-of-function experiments were performed by transfecting LECs for 24 h with miR-19b mimic or inhibitor. RT-qPCR revealed that miR-19b was successfully overexpressed by LECs and reached peak expression with a transfection concentration of 70 pmol/ml (Figure 7C) and miR-19b expression was inhibited and reached a minimum value with a transfection concentration of 200 pmol/ml (Figure 7D). Using these conditions, the experiment showed that upregulation of miR-19b inhibited TGFβRII expression by 50% at the mRNA level (Figure 7E), whereas downregulation of miR-19b increased TGFβRII expression by 1.5-fold at the mRNA level (Figure 7E).

## Discussion

In this study, we found that lymphatic vessel dysfunction occurs in the early stage of atherosclerosis and is paralleled by an increase in miRNA-19b expression. Systemic treatment with EMP^miR-19b^ results in significant increase in miRNA-19b expression in arteries and lymphatic circulation. Increased miRNA-19b levels resulted in decreased distribution of lymphatic vessels along atherosclerotic arteries and also inhibited the transport function and permeability of lymphatic vessels, further aggravating atherosclerosis. *In vitro* experiments verified that miRNA-19b could inhibit LEC migration and lymphangiogenesis, and these effects may be associated with miR-19b–mediated downregulation of TGFβRII. Based on these findings, we speculate that EMP^miR-19b^ exacerbate atherosclerosis, possibly by inducing dysfunction of the lymphatic system in atherosclerosis.

EMPs are one form of endothelial cell-derived extracellular vesicles that contain functional miRNAs, proteins, and other factors. Prior research has shown that EMPs can serve not only as important biomarkers of endothelial dysfunction, but also modulate cell–cell communication, thereby affecting the occurrence and development of cardiovascular diseases (Pernomian et al., 2018). Our previous study demonstrated that systemic delivery of EMP^miR-19b^ accelerated atherosclerosis in carotid arteries, possibly due to excessive vascular inflammation (Li et al., 2018). Consistent with those findings, the results of the present study show that systemic treatment with EMP^miR-19b^ aggravated arterial atherosclerosis in ApoE^-/-^ mice as manifested by higher carotid flow resistance, decreased carotid artery diameter, larger atherosclerotic plaques, and increased instability of plaques with a thinner fibrous cap. These results also agree with the findings of Michael et al. and Ye et al. showing that miR-19b positively regulates inflammatory activation and promotes proinflammatory cytokine secretion (Gantier et al., 2012; Ye et al., 2012).

The lymphatic system is an important circulatory system *in vivo*, and its major functions include regulating the inflammatory response by diverting inflammatory exudation, inflammatory cells, and mediators back to the blood circulation and also mediating lipid metabolism via reverse cholesterol transport (Randolph et al., 2017; Johnson, 2021). Recent studies have reported that with human atherosclerotic carotid arteries, the number of lymphatic vessels, which are distributed mainly in perivascular areas such as the adventitia as well as in intraplaque regions, is associated with the severity of atherosclerotic disease. Some authors also proposed that while the inflammatory environment influences lymphangiogenesis around atherosclerotic vessels, changes in the lymphatic system could reversely affect the process of atherosclerosis (Csanyi and Singla, 2019). However, the specific role of the lymphatic system in atherosclerosis and its mechanism remain unclear. In our previous experiments, systemic injection of CM-Dil-labeled EMP^miR-19b^ in atherosclerotic mice resulted in the accumulation of EMP^miR-19b^ in the perivascular adipose tissue around carotid arteries. This finding suggests that collocation of blood and lymphatic vessels provides a structural basis for the ability of miR-19b to regulate the function and distribution of lymphatic vessels. According to the effects of EMP^miR-19b^ on atherosclerosis observed in that study, we further explored whether atherosclerosis is exacerbated by lymphatic system dysfunction. Our confocal microscopy experiment results indicate that EMP^miR-19b^ inhibited the transport function of lymphatic vessels from the early stage of atherosclerosis, and the permeability and size of lymphatic vessels were also damaged by EMP^miR-19b^, as illustrated by Evans blue staining. In the process of atherosclerotic plaque formation, EMP^miR-19b^ also showed negative effects on the distribution of lymphatic vessels. That these deficiencies in the lymphatic system occurred parallel to the course of the disease means that arterial accumulation of immune cells and changes in inflammatory mediator levels induced by EMP^miR-19b^ treatment may rely on lymphatic function breakdown. Our research has some limitations still, and in the future, we will further elucidate this mechanism by inhibiting miR-19b expression *in vivo* to determine its therapeutic effects on the lymphatic system in atherosclerotic mice.

miR-19b is a member of the miR-17-92 gene cluster, which is highly expressed in human endothelial cells and is involved in the pathological progression of many CVDs. A previous study reported that miR-19b could be a marker of acute coronary syndrome, such as unstable angina. Additional researchers found that upregulation of miR-19b-3p accelerates atherosclerosis by downregulating PGC-1*α* (peroxisome proliferator-activated receptor-gamma coactivator-1alpha), a protein important for maintaining normal mitochondrial function (Xue et al., 2015). As mentioned above, miR-19b may also impair the function of the lymphatic system, which may be another mechanism by which it accelerates atherosclerosis. Our *in vitro* experiments showed that miR-19b mimic could inhibit cell migration and tube formation, whereas miR-19b inhibitor could reverse these effects, confirming that miR-19b may be involved in lymphatic vessel remodeling through changes in LEC migration and lymphangiogenesis. Our findings in LECs are similar to previous observations in vascular endothelial cells, in which miR-19b can also inhibit migration and angiogenesis via TGFβ signaling (Liang et al., 2018). To further elucidate the molecular mechanism of miR-19b, we analyzed possible target genes and selected some related to the lymphatic system for verification. Our results finally confirmed that miR-19b can alter the expression of TGFβRII in LECs, and this receptor has already been determined to be involved in modulate LEC sprouting and branching in recent reports (James et al., 2013). Furthermore, Kunpei F et al. *in vivo* experiments reported that the lymphatic vessels in TGFβRII^-/-^ mice showed abnormal dilation and increased bifurcator. The TGFβ signaling is necessary in maintaining normal structure of lymphatic vessels and lymphatic homeostasis (Fukasawa et al., 2021). Thus, according to our findings and current literature we could concluded that miR-19b may regulate lymphatic system remodeling and dysfunction by affecting LEC migration and lymphangiogenesis, and these effects are likely associated with the expression of TGFβRII.

## Conclusion

In conclusion, the present study provides new mechanistic insight into the role of lymphatic vessels in atherosclerosis and demonstrates the potential role of EMP^miR-19b^ in lymphatic system dysfunction. After systemic EMP^miR-19b^ treatment, miR-19b expression was increased in arteries and lymphatic circulation and led to reduced lymphatic vessel distribution and function, in parallel with atherosclerosis acceleration. Moreover, TGFβRII may mediate the biological effects of miR-19b on the distribution and function of lymphatic vessels.

## Data Availability

The raw data supporting the conclusions of this article will be made available by the authors, without undue reservation.
